# Urban regulatory focus: a new concept linking city size to human behaviour

**DOI:** 10.1098/rsos.171478

**Published:** 2018-05-23

**Authors:** Guy M. Ross, Juval Portugali

**Affiliations:** 1 Porter School of Environmental Studies, Tel Aviv University, Tel Aviv, Israel; 2 Department of Geography and the Human Environment, Tel Aviv University, Tel Aviv, Israel

**Keywords:** promotion, prevention, cities

## Abstract

Why do people in big cities behave differently to those living in small cities? To answer this question, in this paper a new concept of urban dynamics is presented that links city size to human behaviour. The concept has its origins in regulatory focus theory. According to the theory, goal-directed behaviour is regulated by two motivational systems, promotion and prevention. Individuals motivated by promotion goals (growth, accomplishment) focus on winning and tend to take risks, whereas those driven by prevention goals (safety, security) focus on not losing and try to avoid risk. Here we elaborate on the existing literature by linking the theory to the urban context. In our conceptualization, cities are powerful regulatory systems, and as such they impinge upon the way people regulate themselves in the urban space. Evidence from signal detection analysis is provided that supports this concept. The experience of a big-city context intensified both promotion-focused behaviour (a risky bias) for promotion-focused participants and prevention-focused behaviour (a conservative bias) for prevention-focused participants. The experience of a small-city context encouraged the opposite behavioural pattern in both cases. These findings suggest that the urban environment can influence the regulatory focus strategies of an individual in a way that cannot simply be explained by their personal regulatory focus. Specifically, the likelihood of one's behaving in a promotion- or prevention-oriented manner is dependent both on one's chronic regulatory focus and also on the urban context in which one lives. Based on this, we maintain that vibrant cities with a large population and a fast pace of life encourage extreme and polarized behaviours, whereas cities with a smaller population and a slower pace of life encourage more moderate and less polarized behavioural responses, which may explain why people in big cities take more risks, do more business, produce and spend more, and even walk faster.

## 1. Introduction

People living in large cities behave differently to those living in small cities [1]. A key finding is that various urban properties, many of which are related to human behaviour, are power-law functions of population size [2–4]. These specific power-law functions have been shown to have scaling exponents (*β*) that fall into three distinct universality classes. Quantities associated with the social nature of cities (e.g. wealth, innovation) are typified by increasing returns with population size, *β* > 1 (a superlinear regime). Material quantities (e.g. electrical cable length) display economies of scale associated with infrastructure, *β* < 1 (a sublinear regime). Quantities associated with individual human needs (e.g. household electrical and water consumption) are characterized by *β* = 1 (a linear regime). One of the most striking features of this analysis is the numerous indicators that scale superlinearly. Included among these are salaries, income, bank deposits, invention rate (measured by new patents), as well as indicators of negative consequences such as crime rates and disease incidence, and even indicators related to day-to-day city life such as pedestrians' walking speeds [5–13]. All scale superlinearly with population size across nations and over time. This superlinear regime essentially implies that the pace of urban life increases with city size. The question, however, remains as to why behaviour changes with city size. In the present paper we address this question. A new concept of urban dynamics is presented that links city size to human motivation and behaviour. In the discussion which follows, we first introduce the theory of regulatory focus (a prominent and well-established theory of motivation) and its application to behaviour of individuals. We proceed by introducing the idea that the urban context can affect motivation and thus behaviour. Empirical evidence that supports this idea is then presented. Finally, we outline a theoretical framework to account for how cities affect people, and how in turn people affect the city.

## 2. Regulatory focus theory

The main assertion of regulatory focus theory is that individuals' goal-directed behaviour is regulated by two distinct, and independently operating, motivational systems—promotion and prevention [14,15]. The promotion system triggers motivation for accomplishment and growth, is sensitive to positive outcomes, and underlies concerns with advancement from the status quo to a better state. The prevention system, by contrast, triggers motivation for safety and fulfilment of responsibilities, is sensitive to negative outcomes, and underlies concerns with maintenance of the status quo against falling to a worse state. Implicit in this theorizing is the notion that, while all people are driven by both promotion and prevention concerns, individuals differ on the strength of each of the two orientations, and also on their relative strength. Some are more promotion-oriented than others, and some are more prevention-oriented than others. Moreover, one individual may have stronger promotion than prevention concerns (a dominant promotion focus), whereas another may have stronger prevention than promotion concerns (a dominant prevention focus). These differences have implications for the strategies and tactics people apply to pursue goals and also for the means of goal attainment. Individuals with a strong promotion orientation focus on winning, tend to take risks, and maintain a strategic preference for eagerness means of goal attainment (a liberal bias). Those with a strong prevention orientation, by contrast, focus on not losing, try to avoid unnecessary risk, and maintain a strategic preference for vigilant means (a conservative bias). In the current paper, we expand on the existing literature by linking the regulatory focus theory to the urban context.

## 3. Regulatory focus theory and the city

The underlying notion in our theoretical conceptualization is that cities are powerful regulatory systems, and as such they affect the way people self-regulate themselves in the urban space. More specifically, it is proposed that cities influence the regulatory focus strategies embraced by their residents in a way that the likelihood of one behaving in a promotion- or prevention-oriented manner depends both on one's chronic regulatory focus and also on the experience of the urban context, which itself is shaped by the size of the city and the pace of life. The study reported here was designed to test these ideas.

In the first step of our analysis, we distinguished between large and small cities. Metropolitan areas such as New York, Los Angeles and Chicago are large, vibrant and fast-paced urban agglomerations [1,16,17]. They have strong economies that create jobs, increase personal income, and provide opportunities for a high standard of living [18–20]. Cultural attractions, fancy restaurants and an exciting nightlife make these cities centres for culture and entertainment [21–24]. These cities represent an achievement-oriented urban setting with a focus on wealth creation, attainment, growth and creativity [25,26]. They are places to conduct business and pursue careers, to meet people, spend money, entertain, shop, and enjoy arts and culture. But they are also places with relatively high crime rates [27,28]. Now let us take a look at smaller metropolitan areas such as, for example, Altoona, Bangor and Modesto. These are relatively slow-paced cities, and as such they might be a good place for those who prefer a more tranquil kind of urban experience [16,18,21,27,29]. There is evidence suggesting that people who live in large cities behave differently to those living in smaller cities [1,30]. But the question remains, why is this so? To address this issue, a three-step procedure has been applied. First, the participants' chronic regulatory focus was measured. Second, an experimental priming technique was used to make participants temporarily experience either a big-city context or a small-city context. Third, behaviour was assessed by observing the participants’ performance on a signal detection task. It was hypothesized that the experience of a big-city context would encourage participants to engage in promotion-focused behaviours, whereas the experience of a small-city context would discourage participants from displaying promotion-focused behaviours.

## 4. Participants and methods

### 4.1. Participants

Participants comprised 201 undergraduate students from Tel-Aviv University (98 women, 103 men, *M*_age_ = 24.5 years, age range: 22–28). Participation was on a voluntary basis, and students received neither financial reward nor course credits in exchange for taking part. Confidentiality and anonymity were assured throughout.

### 4.2. Participants and procedure

Upon arrival at the laboratory, participants were told that they were part of a study that sought to understand how people memorize information (a cover story), after which they were assigned randomly to one of two experimental conditions, the big-city group or the small-city group. Each group was located in a separate hall. Next, the participants' personal regulatory focus was measured as a chronic variable.

#### 4.2.1. Regulatory focus assessment

Chronic regulatory focus is an established personality trait shaped by a person's accumulated experience in prior goal achievement, and is relatively stable across contexts. As noted earlier, chronic regulatory focus consists of two independent self-regulatory orientations—promotion and prevention. The promotion orientation involves sensitivity to positive outcomes and is characterized by a tendency to approach gains (and avoid non-gains). The prevention orientation involves sensitivity to negative outcomes and is characterized by a tendency to avoid losses (and approach non-losses). To assess chronic regulatory focus, participants completed the regulatory focus questionnaire (RFQ), an experimental tool that was developed to measure dispositional regulatory focus [31], and is commonly used in studies for this purpose [32–36]. The RFQ (see Appendix) contains two psychometrically distinct subscales (a six-item promotion subscale and a five-item prevention subscale) that are used to assess chronic individual differences in regulatory focus. Items in this questionnaire ask how frequently specific events actually occur or have occurred in one's life. Sample items for promotion focus include ‘Compared to most people, are you typically unable to get what you want out of life?’ reverse scored, and ‘Do you often do well at different things that you try?’ forward scored. Sample items for prevention focus include ‘Not being careful has gotten me into trouble at times' reverse scored, and ‘How often did you obey rules and regulations that were established by your parents?’ forward scored. Participants are instructed to answer these questions by indicating a number ranging from 1 (never or seldom) to 5 (very often). The RFQ promotion scores and RFQ prevention scores are then used to determine the respondents' chronic promotion focus and chronic prevention focus, respectively. Having completed the questionnaire, participants proceeded with the signal detection task.

#### 4.2.2. Signal detection task

In a typical signal detection task, a signal is either presented or not presented, and participants have to say either ‘Yes’ (a signal was detected) or ‘No’ (no signal was detected). There are four potential combinations for each trial: (1) hit—a signal was presented and was successfully detected; (2) miss—a signal was presented but it was not detected; (3) false alarm—there was no signal but nevertheless a signal was falsely detected; and (4) correct rejection—there was no signal and no detection. A variation of the signal detection paradigm is the recognition memory task. In this task, participants are first required to memorize nonwords (e.g. ZAJOF). Next, they are shown a mix of previously seen nonwords intermingled with new ones. The task is to discriminate the old from the new, i.e. ‘Have you seen this nonword before?’ with two responses (‘Yes’/‘No’). The dependent measure is the participants' response bias. Saying ‘Yes’ when uncertain is an indicator of a risky bias, whereas saying ‘No’ under uncertainty is an indicator of a conservative bias. Research using the recognition memory paradigm has found that individuals with a strong promotion focus (when compared to those with a strong prevention focus) tend to show a larger response bias on the task [37] (but see [38]). A potential explanation for this is that individuals with strong promotion goals are eager to recognize as many items as possible (maximizing hits and minimizing misses), and this eagerness encourages a liberal approach to risk as reflected in saying ‘Yes’ under uncertainty (a risky bias). By contrast, individuals with strong prevention goals are vigilant not to make errors (maximizing correct rejections and minimizing false alarms), and this vigilance encourages a more cautious approach to risk-taking as reflected in saying ‘No’ when uncertain (a conservative bias).

#### 4.2.3. Task and manipulation

In the present study, a recognition memory task adapted from previous regulatory focus research was used [37,39–41]. In the memorizing phase, participants viewed a sequence of 20 nonwords projected from a computer onto a screen on the wall in front of them. Each nonword was presented for a 2-s period and the complete session lasted 40 s. The instruction was to best memorize the nonwords. Next, participants worked on a filler task for an additional 40 s. Two series of numbers (10 items in each series) were projected onto a screen on the wall. The numbers were presented one after the other, and each for two seconds. The task was to identify the common feature shared by all of the numbers within each series. The first set of numbers was 33,3,30,6,18,9,333,12,24,300, and the second set was 28,35,14,70,7,77,56,700,777,49. It can be seen that the numbers in the first series are multiples of 3, while those in the second series are multiples of 7. The urban context manipulation followed next. Priming was employed to activate the urban context (a big, fast-paced city versus a small, slow-paced city). The big-city group was primed with a video showing large, busy streets, people hurrying and elbowing their way ahead, heavy traffic, high-rise luxury towers, arts and cultural events, street performances, fancy restaurants, a vibrant nightlife scene and, in general, a fast-paced, hectic urban environment. The small-city group was primed with a video showing relatively small, tranquil streets, sparse traffic on the roads, low-rise buildings, people slowly walking while looking at display windows, some hanging out in local cafes, others riding bicycles for recreation and, in general, a slow-paced, leisurely urban setting. The videos were projected onto a large screen on the wall in front of the participants. Having watched the video, participants were given a paper with a list of 40 nonwords, comprising the 20 previously seen ones intermingled with 20 new ones. The task was to decide whether each of the listed nonwords was old or new. Participants were instructed to circle the nonwords they thought were old. No incentive of any kind was offered and there was no time limit. Upon completion of the task, participants were debriefed as to the nature and purpose of the study, thanked for their contribution, and dismissed.

Four points are worth emphasizing before presenting the results. First, the videos showed no features by which to identify the cities. This was to avoid confounds due to preconceptions and stereotypes that could have biased the participants' experience of the city. Second, the soundtrack (background music) was muted to avoid a confounding effect due to priming modality. This was to ensure that the visual information would be the only means of priming. Otherwise, it would have been impossible to rule out the possibility that the music was responsible for any obtained experimental effects. Third, the videos were downloaded from YouTube and no editing was undertaken except for equating the duration of the streams (three minutes each). Fourth, a pilot test was conducted to check the priming manipulation. Sixty-three student volunteers (other than the participants in the main study) took part in a session in which each participant watched two videos (those that were used in the main study). The first video (Video 1) depicted streets full of people and heavy traffic (a big-city context). The second video (Video 2) portrayed small streets and sparse traffic (a small-city context). Next, participants were asked for their opinion about the two cities that were shown in the videos. The questions and the percentage choosing each option are shown. Statistical significance at the 1% level is denoted by*.

**Table:** 

1. In which city is the population larger?		
Video 1	Video 2	$\chi_{\left(1\right)}^{2}=32.143$, *p* < 0.01
[86]*	[14]	
2. In which city is the pace of life slower and more relaxed?		
Video 1	Video 2	$\chi_{\left(1\right)}^{2}=55.254$, *p* < 0.01
[3]	[97]*	

It can be seen that 86% of respondents associated Video 1 with a big-city context, which means that they also associated Video 2 with a small-city context. Furthermore, 97% of respondents associated Video 2 with a slow-paced urban context, which means that they also associated Video 1 with a fast-paced urban context. Taken together, these findings confirmed that both video clips were perceived by the participants as we meant them to be perceived.

## 5. Results

The statistical design consisted of three independent variables, chronic promotion focus and chronic prevention focus (both continuous measured variables assessed with RFQ promotion scores and RFQ prevention scores, respectively) and urban context (a dichotomous variable manipulated between participants—big city versus small city). The dependent variable was response bias on the signal detection task. The response bias indicates the extent to which one is motivated to ensure hits (correctly detecting nonwords from the old list) and avoid misses (fail to detect a nonword that was included in the old list) versus the extent to which one is motivated to ensure correct rejections (correctly identify a new nonword as new) and avoid false alarms (falsely identify a new nonword as a word from the old list). Mathematically, the response bias is calculated with:

$$\text{Br}=\frac{p(\text{false alarm})}{1-p(\text{hit})+p(\text{false alarm})}.$$

Higher values of this measure indicate a more liberal bias as reflected in a tendency to say ‘Yes’ when uncertain about the correct answer—a characteristic of a promotion focus. Lower values indicate a more conservative bias as reflected in a tendency to say ‘No’ when uncertain about the correct answer—a characteristic of a prevention focus [42].

It was hypothesized that under a big-city context, response bias would increase with increasing strength of chronic promotion focus. Under a small-city context, the effect should disappear. A multiple regression analysis was applied to test this hypothesis. There were three predictors, the urban context (dummy coded: 1 for big city, 0 for small city), and chronic promotion focus and chronic prevention focus (measured with RFQ promotion scores and RFQ prevention scores, respectively). All data were standardized prior to analysis. The dependent variable of interest was response bias. Three models were run. Model 1—the three main effects were entered. Model 2—the three two-way interactions were added. Model 3—the three-way interaction was added.

The analysis revealed, as predicted, a positive two-way interaction between urban context and RFQ promotion scores, *β* = 0.217, *t*_(193)_ = 2.41, *p* = 0.017, and a negative two-way interaction (which was not expected at the beginning of this study) between urban context and RFQ prevention scores, *β* = −0.186, *t*_(193)_ = −2.08, *p* = 0.039. Neither main effect reached significance, nor did the two-way interaction between promotion scores and prevention scores, nor did the three-way interaction of urban context, promotion scores and prevention scores. A simple slope analysis was then performed to decompose the two interactions. The analysis of the positive interaction between RFQ promotion scores and urban context revealed a significant effect of RFQ promotion scores in the big-city condition, *β* = 0.039, *t*_(193)_ = 2.872, *p* = 0.005, but a non-significant effect in the small-city condition, *β* = −0.003, *t*_(193)_ = −0.279, *p* = 0.78. The analysis of the negative interaction between RFQ prevention scores and urban context revealed a significant effect of RFQ prevention scores in the big-city condition, *β* = −0.048, *t*_(193)_ = −3.473, *p* = 0.001, but a non-significant effect in the small-city condition, *β* = −0.012, *t*_(193)_ = −1.074, *p* = 0.284. These findings indicated that under a big-city context response bias increased with increasing RFQ promotion scores. Response bias was not related to RFQ promotion scores under a small-city context. Furthermore, under a big-city context response bias decreased with increasing RFQ prevention scores. Response bias was unrelated to RFQ prevention scores under a small-city context.

**Figure 1.**
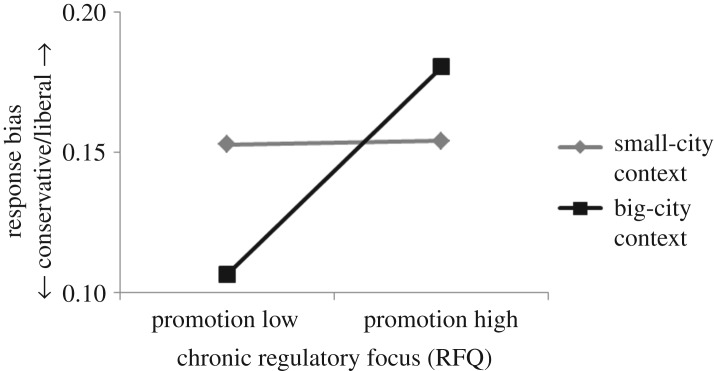
The effect of urban context on promotion-focused participants. A big-city context made participants with high promotion scores display a response pattern more liberal as displayed by those with low promotion scores. A small-city context eliminated the effect. Participants with high promotion scores exhibited a response pattern equally conservative as exhibited by those with low promotion scores.

**Figure 2.**
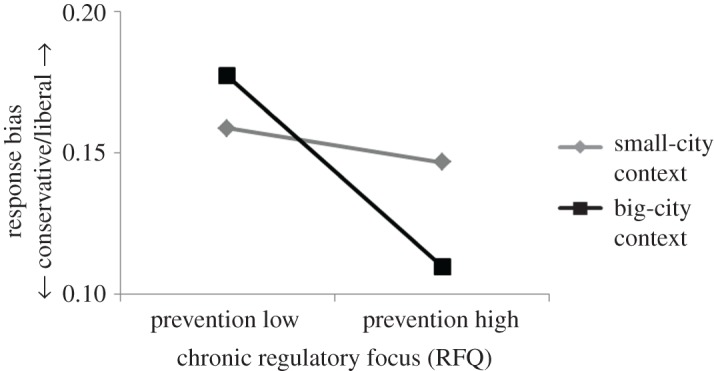
The effect of urban context on prevention-focused participants. A big-city context made participants with high prevention scores adopt a response pattern more conservative as adopted by those with low prevention scores. A small-city context abolished the effect. Participants with high prevention scores demonstrated a response pattern equally liberal as demonstrated by those with low prevention scores.

To illustrate graphically the interaction of urban context and RFQ promotion scores, participants were classified as either high or low on promotion (median split on RFQ promotion scores), and the mean and standard deviation of response bias were then calculated for each group. In the big-city condition, participants high on promotion adopted a more liberal response bias (*M* = 0.18, s.d. = 0.13) than those low on promotion (*M* = 0.11, s.d. = 0.11). In the small-city condition the effect was wiped out. Participants low on promotion were as liberal (*M* = 0.15, s.d. = 0.11) as those high on promotion (*M* = 0.15, s.d. = 0.12) (figure 1). To illustrate the interaction of urban context and RFQ prevention scores, participants were categorized as either high or low on prevention (median split on RFQ prevention scores), and the mean and standard deviation of response bias were calculated for each group. In the big-city condition, participants high on prevention maintained a more conservative response bias (*M* = 0.11, s.d. = 0.1) than those low on prevention (*M* = 0.18, s.d. = 0.14). In the small-city condition the effect was wiped out. Participants low on prevention were as conservative (*M* = 0.16, s.d. = 0.11) as those high on prevention (*M* = 0.15, s.d. = 0.12) (figure 2). Regarding statistical power, a sample size of 76 was calculated to be adequate (desired statistical power level: 0.8, probability level: 0.05, number of predictors: 3, anticipated effect size: 0.15). It is noteworthy that the actual sample size was nearly three times as large as the minimum required. There were no gender-related differences in any of the measurements reported.

**Figure 3.**
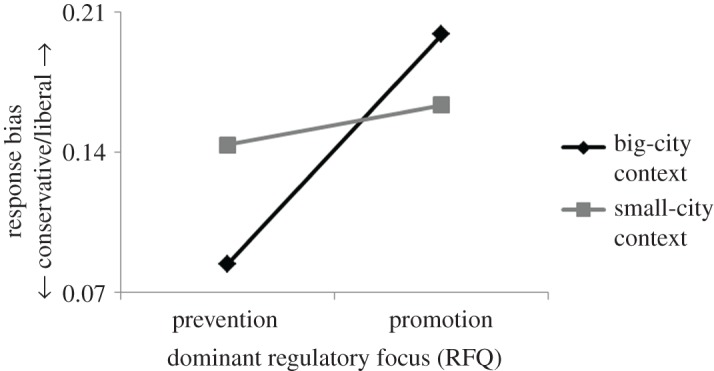
Dominant regulatory focus and the effect of urban context on behaviour. The context of big cities made participants with a dominant promotion focus show a response pattern way more liberal as shown by those with a dominant prevention focus. The context of small cities eliminated the effect. Participants with dominant promotion focus displayed responses equally conservative as displayed by those with dominant prevention focus.

**Figure 4.**
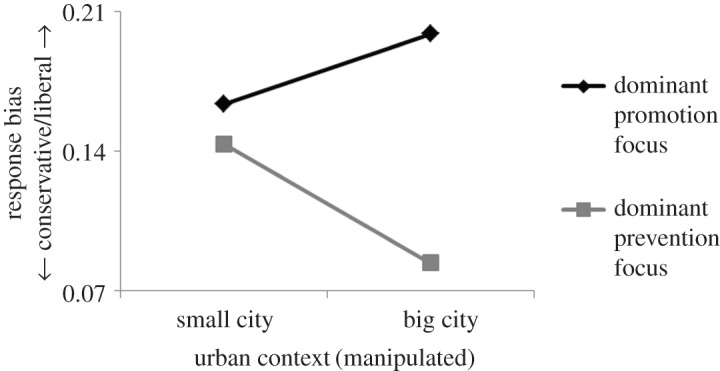
The impact of a change in urban context on behaviour. Under the experience of a small-city context, participants with a dominant promotion focus showed a response pattern as conservative as shown by those with a dominant prevention focus. However, the change in context from small cities to big cities made participants with a dominant promotion focus display an extremely liberal bias, while those with a dominant prevention focus displayed an extremely conservative bias.

Next, the combined effect of dominant regulatory focus and urban context on response bias was analysed. As previously noted, according to regulatory focus theory all individuals are driven by both promotion and prevention concerns. However, people differ on the strength of each of the two orientations and also on their relative strength. Some are more promotion-oriented than others, and some are more prevention-oriented than others. Moreover, some may have a dominant promotion focus (stronger promotion than prevention concerns), whereas others may have a dominant prevention focus (stronger prevention than promotion concerns). The relative strength of the two orientations is particularly important because regardless of the strength of the specific orientations, their relative strength may determine which concerns will emerge as dominant to the individual and drive behaviour. A common way to assess the dominant regulatory focus is by subtracting the participants' RFQ prevention scores from their RFQ promotion scores [43]. Higher scores on this difference measure reflect stronger promotion than prevention concerns (i.e. a dominant promotion focus), whereas lower scores indicate stronger prevention than promotion concerns (i.e. a dominant prevention focus). In keeping with this, a new variable of dominant regulatory focus was created, and then the data were analysed. The statistical design consisted of two independent variables, urban context—a dichotomous variable manipulated between participants (big city versus small city)—and dominant regulatory focus—a continuous calculated variable. The dependent variable of interest was response bias. It was hypothesized that under big-city context, response bias would increase with increasing strength of dominant regulatory focus. Under small-city context, the effect would be abolished. A multiple regression analysis was applied to test this hypothesis. There were two predictors, urban context (dummy coded: 1 for big city, 0 for small city) and dominant regulatory focus (in standardized values), and one dependent variable, response bias (obtained from the Br equation). Two models were run. In Model 1, two main effects were entered. In Model 2, a two-way interaction was added. The analysis revealed, as predicted, a positive two-way interaction of urban context and dominant regulatory focus, *β* = 0.258, *t*_(197)_ = 2.921, *p* = 0.004. Neither main effect reached significance. A simple slope analysis showed a significant effect of dominant regulatory focus in the big-city condition, *β* = 0.055, *t*_(197)_ = 4.243, *p* = 0.000, but a non-significant effect in the small-city condition, *β* = 0.006, *t*_(197)_ = 0.56, *p* = 0.577. These results indicated that response bias only increased with increasing scores of dominant regulatory focus when the urban context reflected large cities with a fast pace of life. Response bias was unrelated to dominant regulatory focus scores when the urban context reflected small cities with a slow pace of life. To illustrate these results graphically, the participants were classified as either predominantly promotion-focused or predominantly prevention-focused (median split on dominant regulatory focus scores). The mean and standard deviation of response bias were then calculated for each group. The data show that under urban context of large, fast-paced cities, the predominantly promotion-focused participants were more than twice as liberal (*M* = 0.2, s.d. = 0.14) as the predominantly prevention-focused ones (*M* = 0.08, s.d. = 0.09). Urban context of small, slow-paced cities completely wiped out the effect. There was no significant difference between the predominantly promotion-focused participants (*M* = 0.16, s.d. = 0.12) and the predominantly prevention-focused ones (*M* = 0.14, s.d. = 0.11) (figure 3). From a slightly different perspective, it can be seen that under the context of small cities, participants with a dominant prevention focus were more or less as liberal (*M* = 0.14, s.d. = 0.11) as those with a dominant promotion focus (*M* = 0.16, s.d. = 0.12). However, the mere transition from a small-city context to a big-city context affected the two groups in completely different ways, encouraging two opposing and polarized behavioural trends. Participants with a dominant promotion focus became more liberal in their behaviour (*M* = 0.2, s.d. = 0.14), whereas those with a dominant prevention focus became more conservative in their behaviour (*M* = 0.08, s.d. = 0.09) (figure 4). Statistical power was again evaluated (same as before except that this time number of predictors: 2). Minimum required sample size was 67 (three times as small as the actual sample size). No gender-related differences were found in any of the measurements.

## 6. Discussion

This study revealed three main findings. First, a positive two-way interaction emerged between urban context and chronic promotion focus. With the urban context of large, fast-paced cities, higher promotion scores (controlling for prevention scores) were positively related to a more liberal response bias. With the urban context of small, slow-paced cities, promotion scores (controlling for prevention scores) were not related to response bias. This specific result suggests that the urban context of large cities made participants with strong promotion goals display a response pattern more liberal as displayed by those with weak promotion goals. The urban context of small cities completely wiped out the effect. Participants with strong promotion goals exhibited a response pattern equally conservative as exhibited by those with weak promotion goals. Second, a negative two-way interaction occurred between urban context and chronic prevention focus. Under the urban context of large, fast-paced cities, higher prevention scores (controlling for promotion scores) were negatively related to a more liberal response bias (i.e. they were positively related to a more conservative response bias). Under the urban context of small, slow-paced cities, prevention scores (controlling for promotion scores) were unrelated to response bias. This particular result implies that the urban context of large cities made participants with strong prevention goals adopt a response pattern more conservative as adopted by those with weak prevention goals. Again, the urban context of small cities completely wiped out the effect. Participants with strong prevention goals demonstrated a response pattern equally liberal as demonstrated by those with weak prevention goals. Here, one could have speculated that if the urban context of large cities intensifies promotion behaviour for individuals with a strong promotion orientation (and the urban context of small cities weakens it), then the urban context of small cities should intensify prevention behaviour for individuals with a strong prevention orientation (and the urban context of large cities should weaken it). But this is not the picture that emerged from our analysis. The first two findings suggest that the urban context of large cities intensifies both promotion- and prevention-oriented behaviour, whereas the urban context of small cities acts in the opposite direction, eliminating the effect in both cases. Third, there was a positive two-way interaction of urban context and dominant regulatory focus. Under the urban context of large, fast-paced cities, higher scores of dominant regulatory focus were positively related to a more liberal response bias (and hence, lower scores on this measure were positively related to a more conservative response bias). Under the urban context of small, slow-paced cities, response bias was not related to dominant regulatory focus scores. This result suggests that the urban context of large cities made participants with a dominant promotion focus show a response pattern more liberal as shown by those with a dominant prevention focus (and hence, it made participants with a dominant prevention focus show a response pattern more conservative as shown by those with a dominant promotion focus). Yet again, the urban context of small cities completely eliminated the effect. Participants with a dominant promotion focus displayed responses that were equally conservative as displayed by those with a dominant prevention focus.

This was *prima facie* evidence that a big-city context is likely to facilitate and enhance both promotion-focused behaviour (risk-taking) among promotion-focused individuals (who are predisposed to take risks) and prevention-focused behaviour (a tendency to avoid risk) among prevention-focused individuals (who are initially risk-averse). A small-city context is likely to encourage the opposite behavioural pattern in both cases. These findings suggest that big, vibrant cities with a large population and a fast pace of life encourage extreme and polarized behaviour, whereas small cities with a slow pace of life encourage more moderate and less polarized behavioural responses.

Of particular importance, the data in the present study came from observations of behaviour in a manipulated urban context. A methodology based on experimental manipulation reduces the possibility of alternative explanations and confounds in the study. Furthermore, as the hypothesized causal direction between urban context and behaviour was directly tested, the methodology provides support for a causal account, namely, that the urban context was responsible for the behavioural differences observed. By demonstrating that the urban context affects the likelihood of one behaving in a promotion- or prevention-oriented manner, the empirical findings reported here strongly support the concept that cities are powerful regulatory systems, and as such they affect motivation and thus the way people regulate their behaviour.

From an external validity perspective, it is noteworthy that in the present work the focus was on North American culture, and more specifically on American cities and the American way of life. Thus, it has to be acknowledged that these findings might be limited and not applicable to other cultures (e.g. Japanese cities and the Japanese way of life). This could be a direction for future research. One more point should be emphasized. In our analysis of cities, we distinguish between a big-city context and a small-city context. The former reflects on large, vibrant cities with a huge population and a fast pace of life, whereas the latter reflects on small cities with a slow pace of life. However, even large, vibrant cities have relaxed and tranquil neighbourhoods, generally in the outer circles of the city. Thus, the urban context discussed in the present work is the one that represents the centre of the city. It is the city centre where differences in pace of life (if they exist) can be seen clearly.

## 7. Conceptual framework

A conceptual framework is presented for modelling the dynamic relationship between the urban environment and the people living in it. It is argued that the way the city affects, and is affected by, the city's residents is a result of a causal interaction between the city residents' regulatory focus and the urban context. Our findings suggest that a big-city context encourages extreme and polarized behaviours. Vibrant cities with a large population and a fast pace of life push individuals in a promotion focus to behave in a more promotion-oriented manner. But these cities also push individuals in a prevention focus to behave in a more prevention-oriented manner. The former effect strengthens the vibrant atmosphere of the city, whereas the latter weakens it. Here is why: when living in a big, vibrant city, promotion-focused individuals, who are predisposed to take risks, take even greater risks (e.g. doing more business, opening more restaurants and bars). This in turn intensifies the pace of life in the urban environment. However, prevention-focused individuals, who are predisposed to avoid risk, show greater risk-aversion, and by following the rationale above, this slows down the pace of life in the urban environment. Thus, in a city there are two competing dynamics, one dominated by the effect of the urban context on promotion-focused individuals, and another dominated by the effect of the urban context on prevention-focused individuals. The interesting question is which of the two dynamics will prevail. Here is what we suggest. Promotion-focused individuals are attracted to and prefer to live in large and vibrant cities, whereas prevention-focused individuals are attracted to and prefer to live in small and slow-paced cities. Thus, in big cities the proportion of promotion-focused inhabitants is likely to be higher than that of prevention-focused inhabitants, which is a sufficient reason to conclude that the prevailing dynamics would be the one dominated by the effect of the urban context on promotion-focused individuals. This topic deserves further investigation, especially the proposition that promotion-focused individuals prefer to live in big cities, whereas prevention-focused individuals prefer to live in small cities. In summary, if the prevailing dynamics in vibrant cities is indeed the one between the urban context and promotion-focused individuals, then this may account for some of the more rampant and interesting psycho-social phenomena in the cities of today. For example, this may explain why in big, vibrant cities people take more risks, do more business, produce and spend more, and even walk faster.

## Data accessibility

All data supporting the results in the article can be found in a couple of files—Data 1, Data 2 (see Step 6: File Upload).

## Appendix. Regulatory focus questionnaire (Higgins *et al.* 2001)

This set of questions asks you HOW FREQUENTLY specific events actually occur or have occurred in your life. Please indicate your answer to each question by circling the appropriate number below it.

**Table:**

1. Compared to most people, are you typically unable to get what you want out of life?					7. Do you often do well at different things that you try?				
1	2	3	4	5	1	2	3	4	5
never or seldom		sometimes		very often	never or seldom		sometimes		very often
2. Growing up, would you ever ‘cross the line’ by doing things that your parents would not tolerate?					8. Not being careful enough has gotten me into trouble at times.				
1	2	3	4	5	1	2	3	4	5
never or seldom		sometimes		very often	never or seldom		sometimes		very often
3. How often have you accomplished things that got you ‘psyched’ to work even harder?					9. When it comes to achieving things that are important to me, I find that I don't perform as well as I ideally would like to do.				
1	2	3	4	5	1	2	3	4	5
never or seldom		sometimes		very often	never true		sometimes true		very often true
4. Did you get on your parents' nerves often when you were growing up?					10. I feel like I have made progress toward being successful in my life.				
1	2	3	4	5	1	2	3	4	5
never or seldom		sometimes		very often	certainly false				certainly true
5. How often did you obey rules and regulations that were established by your parents?					11. I have found very few hobbies or activities in my life that capture my interest or motivate me to put effort into them.				
1	2	3	4	5	1	2	3	4	5
never or seldom		sometimes		very often	certainly false				certainly true
6. Growing up, did you ever act in ways that your parents thought were objectionable?									
1	2	3	4	5					
never or seldom		sometimes		very often					
